# Percutaneous breast biopsies

**DOI:** 10.1590/0100-3984.2018.51.6e1

**Published:** 2018

**Authors:** Selma di Pace Bauab

**Affiliations:** 1 Mama Imagem, São José do Rio Preto, SP, Brazil. E-mail: sbauab@mamaimagem.com. https://orcid.org/0000-0002-1296-2165.

Percutaneous breast biopsies guided by imaging-stereotaxis, ultrasound and magnetic resonance-perhaps represent one of the greatest achievements of breast imaging specialists, just as the breast-conserving surgery introduced by Umberto Veronesi was to surgeons. The use of such biopsies allows physicians to diagnose breast cancer and to plan, in consultation with the patient, the surgery to be performed. In addition, it gives the affected woman time to assimilate a cancer diagnosis, thus providing an important emotional benefit. Percutaneous biopsy also has the advantages of reducing the number of unnecessary surgical procedures and increasing the chance of early diagnosis.

Until recently, the gold standard for evaluating non-palpable abnormalities found on imaging examinations was preoperative imaging-guided needle localization, followed by surgery. Nevertheless, studies in the literature show that, in some cases, the lesion was not removed during surgery, for a variety of reasons^(^^1^^,^^2^^)^. In 1990, Steve Parker published the first article comparing core biopsy and surgical biopsy, demonstrating that the former is a safe, reproducible procedure. In 1995, Parker introduced vacuum-assisted biopsy, in order to obtain a greater amount of tissue, thus improving the correlation between imaging and histopathology^(^^2^^-^^4^^)^. These techniques have been evaluated and compared. Studies correlating percutaneous biopsy and surgical histologic findings have taught us which percutaneous diagnoses are less reliable and warrant surgical excision and which are safe to follow^(^^1^^-^^4^^)^.

Cost analyses have also shown that percutaneous biopsy is more affordable than is surgical biopsy. The use of percutaneous biopsy also reduces hospitalization rates, as well as scarring and deformities of the breast^(^^3^^)^.

In various studies of breast lesions, percutaneous biopsy has been shown to have low false-negative rates. Although it is a safe procedure, it requires protocols to optimize the choice of method for different lesions; the correlation between imaging and histopathological diagnosis of benign and malignant lesions; and the follow-up of patients with benign lesions^(^^1^^,^^5^^)^.

Perhaps the greatest problem related to percutaneous biopsy is not the safety of the method but rather its indication. Many erroneous interpretations of imaging findings, especially ultrasound findings, lead to an increase in the number of inappropriate biopsy indications. On ultrasound, the positive predictive value of percutaneous biopsy ranges from 9% to 12%. However, a study carried out in the American State of Connecticut after the introduction of the breast density notification law showed that with greater experience and confidence in the method, it was possible to increase the positive predictive value from 6.5% to 25%^(^^5^^)^.

The study published in this issue of Radiologia Brasileira, entitled “Magnetic resonance imaging-guided vacuum-assisted breast biopsy: experience and preliminary results of 205 procedures”^(^^6^^)^, carried out at a private clinic, discusses all of the requirements for this type of biopsy: a generally appropriate indication, the authors reporting a malignancy rate of 21%; use of the ideal method (vacuum-assisted biopsy), given that the lesions in their sample were seen only on magnetic resonance imaging, vacuum-assisted biopsy being the appropriate approach in such cases; and experience with the method. Magnetic resonance imaging-guided biopsy is not readily available in Brazil, mainly because of its cost. This was an important study, because it brings this procedure closer to our reality, with results comparable to those of the literature, and shows that it can be successfully reproduced. In their study, Carneiro et al.^(^^6^^)^ also evaluated the correct use of the BI-RADS classification in magnetic resonance imaging, showing once again the importance of image analysis for its classification and for the indication of further investigation.

It is important to monitor patients diagnosed with benign lesions. As we mentioned at the outset, percutaneous biopsy has revolutionized the specialty of breast imaging. However, it requires commitment on the part of the radiologist and a multidisciplinary approach involving the pathologist and the surgeon.
